# SARS-CoV-2 papain-like protease plays multiple roles in regulating cellular proteins in the endoplasmic reticulum

**DOI:** 10.1016/j.jbc.2023.105346

**Published:** 2023-10-12

**Authors:** Mei Yang, Jennifer Mariano, Rebecca Su, Christopher E. Smith, Sudipto Das, Catherine Gill, Thorkell Andresson, Jadranka Loncarek, Yien Che Tsai, Allan M. Weissman

**Affiliations:** 1 Cancer Innovation Laboratory, Center for Cancer Research, National Institutes of Health, Frederick, Maryland, USA; 2 Protein Characterization Laboratory, Cancer Research Technology Program, Frederick National Laboratory for Cancer Research, Frederick, Maryland, USA

**Keywords:** deubiquitinase, Nsp, innate immunity, interferon, ISG15, proteolysis, deubiquitination, deubiquitinating enzyme, ubiquitin, COVID, virology, cholesterol, lipid biosynthesis, virulence factor, cell biology

## Abstract

Nsp3s are the largest nonstructural proteins of coronaviruses. These transmembrane proteins include papain-like proteases (PLpros) that play essential roles in cleaving viral polyproteins into their mature units. The PLpro of SARS-CoV viruses also have deubiquitinating and deISGylating activities. As Nsp3 is an endoplasmic reticulum (ER)-localized protein, we asked if the deubiquitinating activity of SARS-CoV-2 PLpro affects proteins that are substrates for ER-associated degradation (ERAD). Using full-length Nsp3 as well as a truncated transmembrane form we interrogated, by coexpression, three potential ERAD substrates, all of which play roles in regulating lipid biosynthesis. Transmembrane PLpro increases the level of INSIG-1 and decreases its ubiquitination. However, different effects were seen with SREBP-1 and SREBP-2. Transmembrane PLpro cleaves SREBP-1 at three sites, including two noncanonical sites in the N-terminal half of the protein, resulting in a decrease in precursors of the active transcription factor. Conversely, cleavage of SREBP-2 occurs at a single canonical site that disrupts a C-terminal degron, resulting in increased SREBP-2 levels. When this site is mutated and the degron can no longer be interrupted, SREBP-2 is still stabilized by transmembrane PLpro, which correlates with a decrease in SREBP-2 ubiquitination. All of these observations are dependent on PLpro catalytic activity. Our findings demonstrate that, when anchored to the ER membrane, SARS-CoV-2 Nsp3 PLpro can function as a deubiquitinating enzyme to stabilize ERAD substrates. Additionally, SARS-CoV-2 Nsp3 PLpro can cleave ER-resident proteins, including at sites that could escape analyses based on the established consensus sequence.

Severe acute respiratory syndrome coronavirus 2 (SARS-CoV-2) is the viral pathogen responsible for the COVID-19 pandemic. Nonstructural protein 3 (Nsp3), the largest nonstructural coronavirus (CoV) protein, is synthesized as a polyprotein with other Nsps. Nsp3s include domains that have a variety of functions related to viral pathogenesis and have two transmembrane domains that localize them to the endoplasmic reticulum (ER) when expressed alone or during the initial phases of infection. Topologically, all but a short region that binds Nsp4 faces the cytosol. Nsp3 and Nsp4, together with Nsp6, are required to form the double membrane ER-derived SARS-CoV replication/transcription complex (1, 2, 3, 4).

The Nsp3s of the genus beta-CoV, which comprises SARS-CoV-2 as well as SARS-CoV-1 and Middle East respiratory syndrome (MERS)-CoV, include a single papain-like protease (PLpro) composed of a ubiquitin-like domain and protease domain. PLpro, which is situated N terminal to the two Nsp3 transmembrane domains, plays an essential role in the cleavage of CoV polyproteins into their constituent mature proteins, releasing Nsp1, Nsp2, and Nsp3 itself (1). Other polyprotein cleavages are carried out by the Nsp5 3CLpro domain (5). For MERS, SARS-CoV-1, and SARS-CoV-2, the cleavage consensus site is L_(P4)_X_(P3)_G_(P2)_G_(P1)_, using the numbering system of Schechter and Berger (6), where cleavage occurs after the P_1_ Gly. An exception is the Nsp3-Nsp4 cleavage site in MERS where an Ile is found at P_4_ (7). In addition to its role as an endoprotease for viral polyproteins, PLpro of some coronaviruses, including those noted above, can also remove both polyubiquitin chains and ISG15 from proteins (8, 9, 10). These related protein modifiers are linked to proteins through their C termini, which both terminate with LRGG. SARS-CoV-2 PLpro has a preference for removing ISG15 (11, 12, 13, 14), while SARS-CoV-1 PLpro has higher activity toward K48-linked diubiquitin (15, 16, 17, 18). These differences have been ascribed to diminished activity toward ubiquitin linkages in SARS-CoV-2 PLpro due to alterations in its S1 site (19).

For SARS-CoV-1 and SARS-CoV-2, studies on ubiquitination have focused on antagonistic roles that PLpro might play as a deubiquitinating enzyme (DUB) toward activators of innate immunity mediated by a type 1 interferon (IFN) response. Here, ubiquitination of multiple proteins plays critical roles in activating signaling through K63 and other polyubiquitin linkages, rather than by targeting these proteins for degradation. In several studies examining either transmembrane or soluble cytoplasmic SARS-CoV-1 PLpro, attenuation of the IFN response was found to be independent of PLpro catalytic activity and attributed to disruption of protein–protein interactions (20, 21, 22, 23). In a study assessing the effects of cytosolic SARS-CoV-2 PLpro on IFN activation, a requirement for DUB activity was found for some, but not other, critical mediators of this response (24). Another recent study found catalytic activity to be required for cytosolic SARS-CoV-2 PLpro to suppress an IFN response induced by stimulator of interferon genes (STING) overexpression (25), which undergoes activating ubiquitination in the ER. This contrasts with previous work employing a transmembrane SARS-CoV-1 PLpro, where decreased STING activation and ubiquitination was independent of PLpro catalytic activity (21, 22). It remains unclear whether cytosolic *versus* transmembrane forms of the same PLpro would impact requirements for PLpro DUB activity and what the overall importance of membrane tethering is to immune suppression by SARS-CoV-2 PLpro. Nevertheless, there is substantial data to suggest that catalytic activity is dispensable for PLpro to oppose activation of multiple signaling molecules integral to the innate immune response.

In addition to roles in the IFN response, the ER is a hub for other signaling, including cellular stress responses and pathways leading to cholesterol and fatty acid biosynthesis. Proteins in these pathways and others are regulated in part by a ubiquitin and proteasome-dependent homeostatic process known as ER-associated degradation (ERAD), which is also central to ER quality control (26, 27, 28, 29). Activation of lipid biosynthesis occurs through the trafficking of sterol regulatory element–binding protein (SREBP) -1 and -2 bound to SREBP cleavage–activating protein (SCAP) to the Golgi. This takes place upon their release from insulin–induced protein (INSIG) –1 and -2 in the ER in response to stimuli including cholesterol depletion (30, 31). INSIG-1, in particular, has been extensively characterized as a short-lived ERAD substrate (31, 32, 33, 34, 35, 36). In the Golgi, SREBPs undergo endoproteolytic cleavage by S1P and S2P, leading to release of their N-terminal domains (NTDs), which constitute the active transcription factors (30, 37, 38). SREBPs have also been reported to be targeted for ERAD (39, 40) and to be essential for coronavirus propagation (41).

Despite the ER localization of Nsp3, there has been little investigation into a possible role for PLpro in modulating ERAD. We therefore examined the effects of transmembrane forms of SARS-CoV-2 PLpro on exogenously expressed INSIG-1, SREBP-1, and SREBP-2. Our findings reveal that catalytically active transmembrane PLpro impacts these proteins in different ways by decreasing ubiquitination and, unexpectedly, by endoproteolytic cleavage including at noncanonical sites. In contrast to the type I IFN response, all the effects we observe require the catalytic activity of PLpro and most require ER membrane anchoring of the enzyme. Overall, our observations suggest that PLpro potentially plays an underappreciated important role in determining the fate of ER proteins and proteins that partition into the replication/transcription complex during SARS-CoV-2 infection.

## Results

### SARS-CoV-2 Nsp3 localizes to the ER

**Figure 1:**
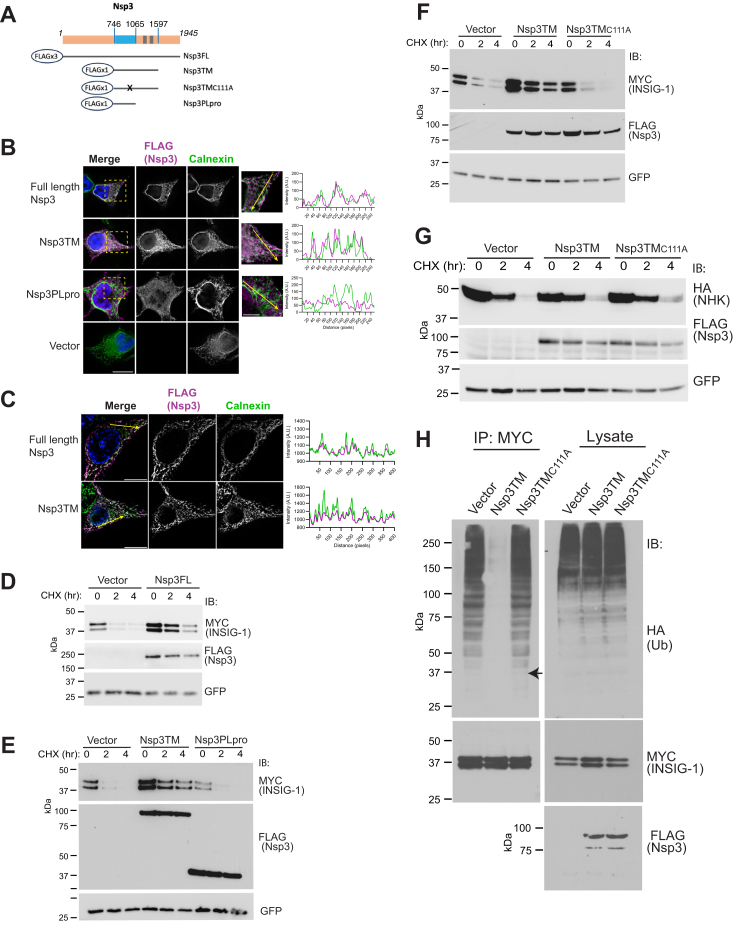
**Transmembrane SARS-CoV-2 Nsp3 localizes to the ER and delays degradation of INSIG-1.***A*, schematic representation of forms of FLAG-tagged Nsp3 used in this study. Amino acid numbers shown at the *top* correspond to the C termini of the constructs and the boundaries of PLpro. Transmembrane domains are indicated in *gray*, PLpro in *blue*. Mutation in the active site Cys (aa 111 of PLpro) is indicated by “x.” *B*, HEK293T cells were transfected with the indicated N terminally FLAG-tagged forms of Nsp3. Samples were evaluated for colocalization of Nsp3 with endogenous Calnexin by wide field microscopy. The scale bar in *lower left panel* corresponds to 10 μm; and the scale bar in *lower left panel* of magnified images corresponds to 5 μm. Assessment of colocalization was carried out as described in Experimental procedures and plotted as a function of distance from the beginning of the arrow, intensity is in arbitrary units. *C*, HEK293T cells transfected with either full-length Nsp3 or Nsp3TM were assessed for colocalization with Calnexin by SIM. The scale bar (*left panels*) corresponds to 10 μm, assessment of colocalization as in (*B*). *D*, HEK293T cells were transfected with full-length Nsp3, which includes a triple FLAG tag, or with vector control (Vector) along with INSIG-1 and assessed by cycloheximide (CHX) chase. *E*, HEK293T cells were transfected with Nsp3TM or soluble PLpro (Nsp3PLpro), both of which include a single N-terminal FLAG tag, and assessed as in (*D*). *F*, HEK293T cells were transfected with Nsp3TM, catalytically inactive NspTM_C111A_, or vector control and assessed as in (*D*). *G*, HEK293T cells were transfected with the indicated forms of Nsp3 and the Hong Kong Null variant of alpha-1 anti-trypsin (NHK). Degradation was assessed as in (*D–F*). *H*, HEK293T cells were transfected with INSIG-1, HA-tagged ubiquitin, and either Nsp3TM, Nsp3TM_C111A_, or vector control. Cells were treated overnight with MG132 to inhibit proteasomal degradation of ubiquitinated proteins prior to lysis. Samples were immunoprecipitated with anti-MYC to assess INSIG-1 ubiquitination, equal amounts of each lysate were also assessed for total cellular ubiquitination. In (*D–G*), GFP serves as a transfection efficiency control. ER, endoplasmic reticulum; NHK, Null Hong Kong; Nsp, nonstructural protein; PLpro, papain-like protease ; SARS-CoV, severe acute respiratory syndrome coronavirus; SIM, structured illumination microscopy; TM, transmembrane.

Full-length Nsp3 from coronaviruses are known to localize to the ER prior to formation of the viral replication/transcription complex, which itself incorporates ER membrane and ER proteins (2, 3, 4). To confirm ER localization for SARS-CoV-2 Nsp3, we assessed N-terminal FLAG-tagged full-length Nsp3 (Nsp3FL) and two truncated forms of Nsp3 (Fig. 1*A* -schematic). Both truncations begin at the PLpro. One terminates immediately after the PLpro domain (Nsp3PLpro), while the other extends through the two predicted transmembrane domains and an adjacent amphipathic helix (Nsp3TM) (1). Both Nsp3FL and Nsp3TM colocalize with calnexin at the ER, while soluble Nsp3PLpro does not (Fig. 1, *B* and *C*).

### Transmembrane forms of Nsp3 increase levels of the ERAD substrate, INSIG-1

We asked whether Nsp3 affects levels of transfected INSIG-1, which is a multitransmembrane ERAD substrate. Nsp3FL markedly increases the level of exogenously expressed INSIG-1 and delays its loss during a cycloheximide (CHX) chase in HEK293T cells (Fig. 1*D*). Nsp3TM also results in increased levels of INSIG-1, while the cytosolic Nsp3PLpro does not (Fig. 1*E*). Similarly, unlike Nsp3TM, catalytically inactive Nsp3TM in which Cys111 of PLpro is mutated to Ala (Nsp3TM_C111A_) does not increase INSIG-1 levels in HEK293T cells (Fig. 1*F*), while both the WT and mutant forms of Nsp3TM coimmunoprecipitate INSIG-1 (Fig. S1*A*). Similar PLpro activity–dependent INSIG-1 stabilization is observed in HeLa cells (Fig. S1*B*). In contrast to INSIG-1, the level of the Null Hong Kong (NHK) variant of alpha1-antitrypsin, a luminal ER protein that is extensively employed as a model ERAD substrate (36, 42, 43, 44), is not increased by Nsp3TM (Fig. 1*G*) Importantly, active Nsp3TM but not Nsp3TM_C111A_ results in a marked decrease in INSIG-1 ubiquitination without altering total cellular ubiquitination, as assessed after proteasome inhibition to prevent INSIG-1 degradation (Fig. 1*H*). These findings are consistent with transmembrane PLpro functioning as a DUB for INSIG-1.

### Nsp3 differentially affects SREBP-1 and SREBP-2

**Figure 2:**
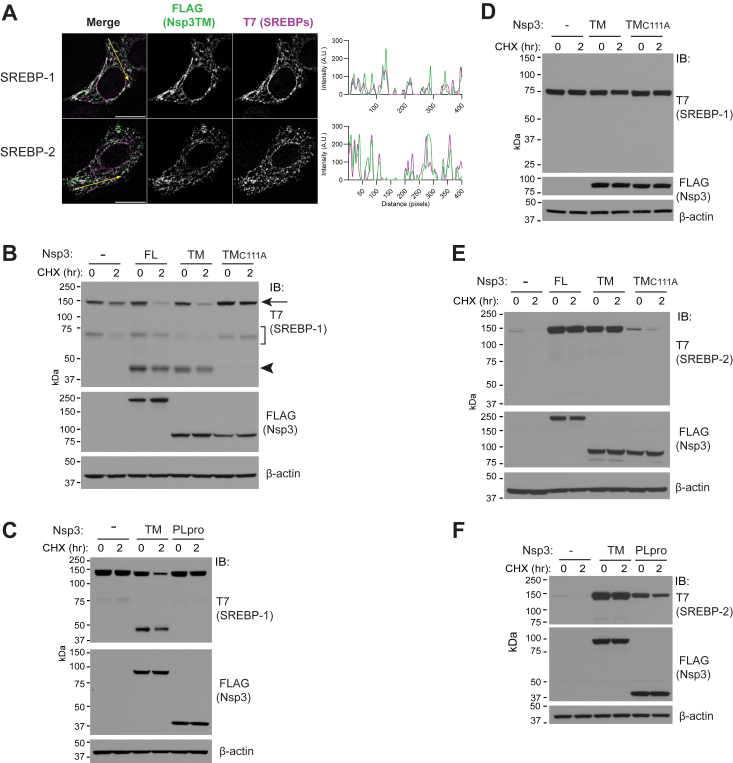
**Differential effects of Nsp3 on SREBP-1 and SREBP-2.***A*, HEK293T cells were cotransfected with plasmid encoding T7 epitope-tagged SREBP-1 (isoform 1) or SREBP-2 and Nsp3TM and imaged by SIM. The scale bar (*left panels*) corresponds to 10 μm, assessment of colocalization as in Figure 1*B*. *B* and *C*, HEK293T cells were transfected with SREBP-1 and either vector control (−) or the indicated forms of Nsp3 and assessed by immunoblotting for SREBP-1 levels and degradation after 2 h of CHX. In (*B*), bracket indicates migration of N-terminal fragments of SREBP-1 that are inconsistently observed regardless of Nsp3 coexpression, *arrow* denotes migration of full-length SREBP-1, *arrowhead* indicates Nsp3-dependent species. *D*, HEK293T cells were transfected with the N-terminal domain (NTD) of SREBP-1, which includes the active transcription factor form of the protein (aa 1–520) and with the indicated forms of Nsp3 and analyzed as in (*C*). *E* and *F*, HEK293T cells were transfected with SREBP-2 and indicated forms of Nsp3 and analyzed as in (*B* and *C*). In (*B–F*), cells were split after transfection, β-actin serves as an equal loading control. CHX, cycloheximide; Nsp, nonstructural protein; SREBP, sterol regulatory element–binding protein; SIM, structured illumination microscopy; TM, transmembrane.

We next evaluated full-length ER-localized SREBP-1 and SREBP-2. Like Nsp3, SREBPs have two closely spaced transmembrane domains, with both their N and C termini facing the cytosol. Using N-terminal T7-tagged forms, we established that exogenously expressed SREBP-1 and SREBP-2 both colocalize with Nsp3 (Fig. 2*A*). We then examined the effects of coexpression of different forms of Nsp3 on SREBP stability in HEK293T cells. When SREBP-1 is expressed with either Nsp3FL or Nsp3TM, but not with inactive Nsp3TM_C111A_, we observe an unexpected acceleration in the loss of SREBP-1 over 2 h of treatment with CHX (Fig. 2*B*, upper panel arrow). Overexpression of SREBP-1 also results in variable amounts of T7 immunoreactive species in the 70 kDa range (Fig. 2*B*, upper panel bracket). These have been observed by others (45) and may represent SREBP-1 that has translocated to the Golgi and been cleaved by S1P and/or S2P. Most strikingly, however, is that both Nsp3FL and Nsp3TM result in T7 immunoreactive species migrating at ∼45 kDa (Fig. 2*B*, arrowhead). No evidence for these ∼45 kDa species, or of accelerated loss of full-length SREBP-1, is apparent with soluble cytoplasmic Nsp3PLpro (Fig. 2*C*). As full-length SREBP-1 in Figure 2, *B* and *C* is epitope-tagged with a C-terminal E-tag, we assessed a form lacking this tag and found it similarly affected by Nsp3TM (Fig. S2*A*). Except where indicated, E-tagged SREBP-1 is used in the rest of this study. Nsp3TM coexpression had no effect on the soluble NTD of SREBP-1, which corresponds to the active nuclear transcription factor (30) (Figs. 2*D* and S2*B*). In striking contrast to SREBP-1, both Nsp3FL and catalytically active Nsp3TM increase the level and inhibit the degradation of SREBP-2 (Fig. 2*E*). This increase is not observed with Nsp3TM_C111A_. Soluble Nsp3PLpro also results in some increase in the level of SREBP-2, albeit to a lesser extent than transmembrane Nsp3TM (Fig. 2*F*).

### Transmembrane forms of Nsp3 PLpro cleave SREBP-1 at multiple sites

**Figure 3:**
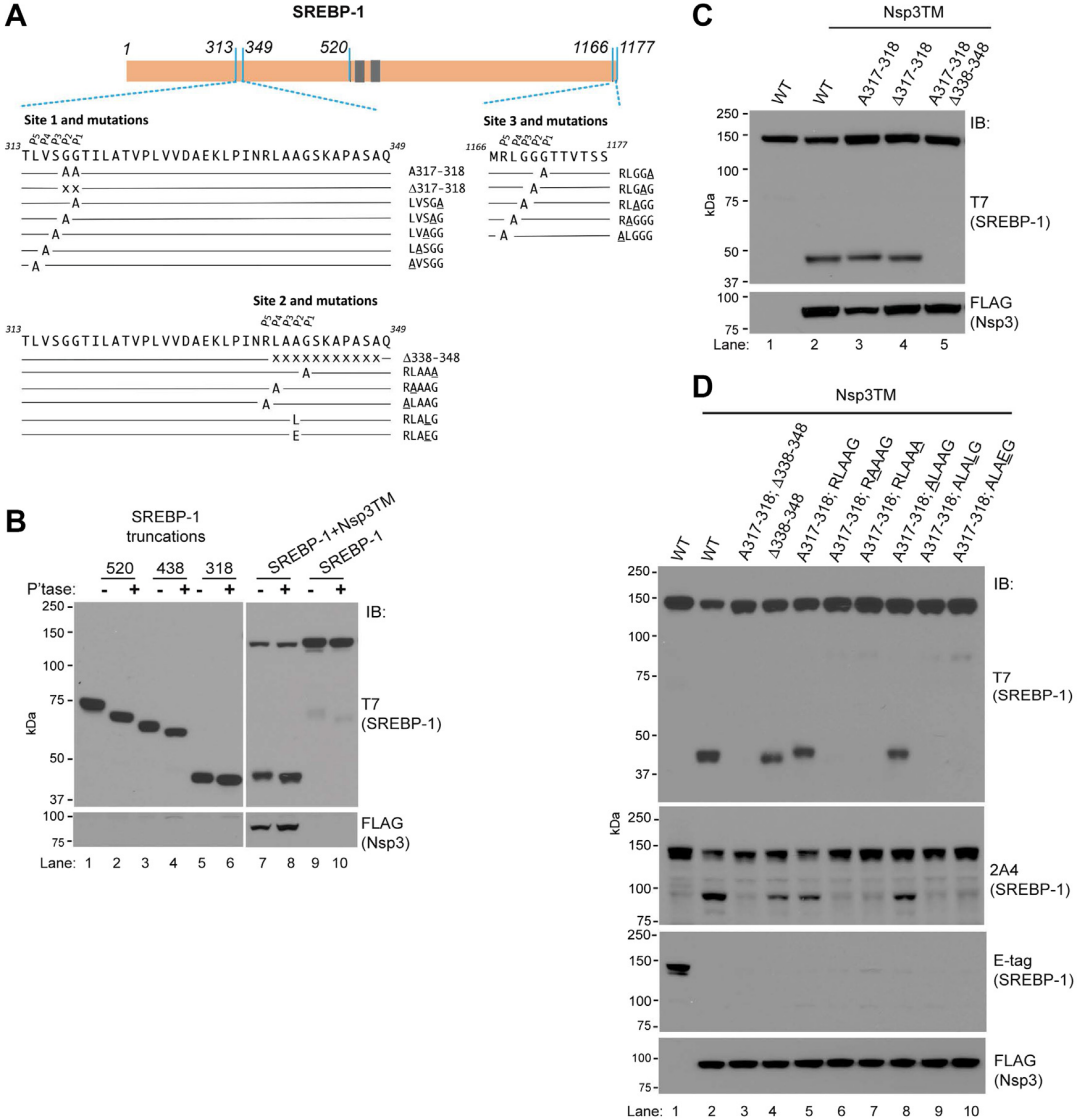
**SREBP-1 is cleaved at multiple sites by Nsp3.***A*, *top*, linear representation of untagged SREBP-1 (isoform 1). Transmembrane domains (TMD) are indicated in *gray*, 520 is the S2P site of cleavage that results in the N-terminal domain (NTD) active transcription factor. Regions corresponding to aa 313 to 349 and 1166 to 1177 (C terminus of SREBP-1) are shown below with mutations and deletions in this study denoted by the single letter of the substituted amino acid or by “x” in the case of deletions. Shorthand terminology for mutations and deletions in each region of the protein are to the *right* (point mutations are *underlined*). Nomenclature for amino acids in proposed cleavage sites (P_1_-P_5_) is from Schechter and Berger (6). *B*, HEK293T cells were transfected with truncated forms of SREBP-1 alone (numbers indicate terminal amino acid included in sequence) or with full-length SREBP-1 with or without cotransfection of Nsp3TM. After treatment with phosphatase (P’tase) samples were resolved by SDS-PAGE and immunoblotted. *C*, HEK293T cells were transfected with or without Nsp3TM and either WT SREBP-1 (lanes 1 and 2) or the indicated mutations or deletions (lanes 3–5) and immunoblotted. *D*, HEK293T cells were transfected as in (*C*). Lanes 5 to 10 include the SREBP-1 A317-318 mutation with either the WT site 2 (lane 5) or single amino acid substitutions in the region of site 2 from 337 to 341 (lane 6–10). See schematic (*A*) “site 2 and mutations.”Nsp, nonstructural protein; SREBP, sterol regulatory element–binding protein; TM, transmembrane.

To begin to understand the molecular basis for the Nsp3-dependent appearance of ∼45 kDa forms of SREBP-1, we compared their migration to SREBP-1 truncations. Both SARS-CoV-2 and SARS-CoV-1 PLpro cleave SARS-CoV polyproteins after the second of two Gly residues (diGly). Additionally, in SARS-CoV polyproteins, a Leu is invariably found in the P_4_ position (LXGG) (7). There are a number of diGlys in the N-terminal half of SREBP-1. Because of the predicted molecular weights, we generated truncations following diGlys at aa 437-438 and 317-318, despite neither having a Leu at P_4_. For comparison, we also evaluated the NTD, which ends at aa 520 (Fig. 3*A* - schematic). Both with and without phosphatase treatment, the 318 truncation closely approximates the migration of the Nsp3-dependent cleavage products (Fig. 3*B*, lanes 5–8). Notably, this truncation lacks the basic helix-loop-helix leucine zipper motif, which is required for the transcriptional activity of the NTD (30, 31, 46).

To further evaluate this cleavage, we either mutated the diGly at 317 and 318 to Ala (A317-318) or deleted these amino acids (Δ317-318) (Fig. 3*A* – site 1 and mutations). Neither of these eliminated the Nsp3-dependent species (Fig. 3*C* lanes 3 and 4). We therefore entertained the possibility of more than one cleavage site in this region of SREBP-1, but the closest diGly are over 100 amino acids in either direction at aa 200-201 and the aforementioned aa 437-438. However, there is a AlaGly 23 amino acids from aa 318 at residues 340-341, which includes a Leu in the predicted P_4_ position (Fig. 3*A* – site 2 and mutations). A deletion of amino acids 338-348 (Δ338-348) in combination with the A317-318 mutation resulted in loss of the lower molecular weight species (Fig. 3*C* lane 5 and Fig. 3*D* upper panel lane 3). Importantly, Δ338-348 does not itself result in loss of these forms (Fig. 3*D* lane 4). These findings support two sites of cleavage in proximity. This is further supported by comparing the relative migration of these N terminal–containing species in the Δ338-348 deletion and the A317-318 mutation (Fig. 3*D* lanes 4 and 5), each of which would leave only one of the two sites intact.

The requirements for cleavage in the region encompassing Gly341, hereafter designated site 2, was examined by point mutation of individual residues to Ala in the context of SREBP-1 that also includes the A317-318 mutation, in what we designate as site 1. We compared these point mutations to SREBP-1 having the A317-318 mutation but retaining the WT site 2 sequence (Fig. 3*D* top panel lanes 5–10, mutations to Ala in lanes 6-10 underlined—see schematic Fig. 3*A*). This demonstrates that the predicted cleavage site Gly (P_1_) is required for cutting, as is the Leu in P_4_, while the Arg at P_5_ is not (Fig. 3*D* lanes 7, 6, and, 8, respectively). These findings implicate this region as a site of cleavage but suggest adequate flexibility to allow for Ala in P_2_. To determine whether the amino acid in the predicted P_2_ position is of significance, the Ala was mutated to either Leu, a larger hydrophobic amino acid, or to Glu, an acidic residue. Both resulted in a loss of cutting (Fig. 3*D* lanes 9–10). Thus, site 2 represents a noncanonical Nsp3 cleavage site. Further confirmation of two sites is shown by comigration of cleavage products of SREBP-1 having only site 1 or site 2 intact with corresponding truncations at each of these sites (Fig. S3*A*). Similar results are observed in HeLa cells (Fig. S3*B*).

In Figure 3*D*, we also immunoblot with the SREBP-1 monoclonal antibody-2A4 (47). This antibody reveals immunoreactive species at ∼90 kDa in lanes where cleavage products are detected by T7 blotting. These bands are complementary in size to those seen with T7 and therefore represent the region of SREBP-1 C terminal to sites 1 and 2. An E-tag immunoblot is also shown in Figure 3*D* (see Fig. S3*C* for complete E-tag autoradiograph from Fig. 3*D*). Strikingly, immunoreactivity with this C-terminal tag is almost completely lost with Nsp3 co-expression regardless of site 1 and 2 mutations. A likely explanation for this lies in the sequence RLGGG near the C terminus of SREBP-1 (1167–1171; see Fig. 3*A* schematic, site 3 and mutations).

**Figure 4:**
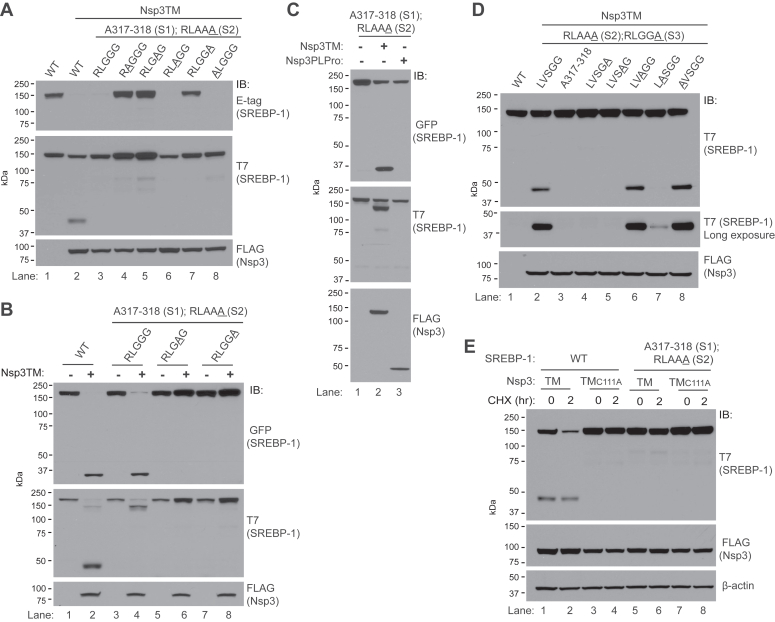
**Assessment of sites 1 and 3 of SREBP-1.***A*, HEK293T cells were transfected with either WT SREBP-1 in lanes 1 to 2 or mutants that abrogate cutting in site 1 (S1) and site 2 (S2) (A317–318 and RLAAA) in lanes 3 to 8, with either the native sequence (lane 3) or the indicated point mutations (*underlined*) in the C-terminal cleavage site (site 3) encompassing aa 1168 to 1172. Lanes 2 to 8 were cotransfected with Nsp3TM. *B*, HEK293T cells were transfected with either Nsp3TM or vector control (−) and C-terminal GFP fusions of SREBP-1. SREBP-1-GFP used in lanes 3 to 8 all include the indicated mutations in the first and second sites as in (*A*) and either the native sequence (lanes 3–4) or the indicated point mutations in site 3 (lanes 5–8). *C*, HEK293T cells were transfected with SREBP-1-GFP having WT site 3 (RLGGG) and mutated in site 1 and site 2 as in (*B*), and the indicated forms of Nsp3 or vector control (−), and immunoblotted as indicated. *D*, HEK293T cells were transfected with plasmid encoding WT SREBP-1 (lane 1) or forms that are cleavage resistant in site 2 (S2) and site 3 (S3) (lanes 2–8) and having the indicated sequences in site 1, and cotransfected with Nsp3TM as indicated. *Middle panel* is a long exposure of the relevant area of the T7 blot showing residual cleavage of the LVSGG to LASGG mutation. *E*, HEK293T cells were transfected with WT SREBP-1 (lanes 1–4) or SREBP-1 that is cleavage resistant in sites 1 and 2 (lane 5–8) and either Nsp3TM or catalytically inactive Nsp3TM_C111A_ and assessed for loss of the full-length protein by CHX chase. In (*E*), cells were split after transfection, β-actin serves as an equal loading control. CHX, cycloheximide; Nsp, nonstructural protein; SREBP, sterol regulatory element–binding protein; TM, transmembrane.

To investigate the loss of E-tag immunoreactivity, systematic mutations to Ala of aa 1167-1171, referred to now as site 3, in the context of mutations in site 1 and site 2, were undertaken. Mutation of either Gly1171 at P_1_ or Leu1168 at P_4_ to Ala results in retention of high-molecular weight E-tag reactivity as does mutation of the P_2_ Gly to Ala (Fig. 4*A*, compare lanes 7, 4, and 5 to WT site 3 lane 3). The loss of cutting with Ala in P_2_ is of note given the naturally occurring Ala in this position in site 2. As with site 2, the P_5_ Arg is dispensable for site 3 proteolysis (Fig. 4*A*, lane 8). To verify our findings, we generated T7-tagged forms of SREBP-1 with C-terminal GFP tags. An ∼35 kDa Nsp3TM-specific anti-GFP reactive species is observed with either WT SREBP-1 or with mutations in sites 1 and 2. Mutation of either Gly1171 or Gly1170 to Ala results in loss of this species (Fig. 4*B*, compare lanes 2 and 4 to lanes 6 and 8). No cleavage is observed at site 3 with expression of the nontransmembrane Nsp3PLpro (Fig. 4*C* lane 3). Similarly, Nsp3TM_C111A_ fails to result in cleavage, while cutting is observed with Nsp3FL (Fig. S4*A*).

We next evaluated site 1 of SREBP-1 in which site 2 and site 3 are not cleavable. As with site 3, mutation of Gly to Ala in either the predicted P_1_ or P_2_ position results in loss of cutting, while neither Ser in P_3_ nor the Leu in P_5_ is required (Fig. 4*D*, compare lanes 4 and 5 to lanes 6 and 8). Site 1 has a Val rather than Leu in the predicted P_4_ position. Mutation of this Val to Ala largely eliminates cleavage (Fig. 4*D* lane 7), although some residual cutting is apparent on long exposure. Together with Val being native to this position, this suggests that site 1 has a degree of flexibility beyond the canonical LXGG.

Establishing that there are only three sites, and determining how they can each be interrupted, allowed us to use mass spectrometry (MS) as additional proof for the noncanonical sites. Constructs were generated and mutated in the canonical site 3 and either site 1 or site 2 (single Gly to Ala mutations at 318 or 341), thus leaving only one site intact. This allowed for immunoprecipitation after cotransfection of Nsp3TM from either the N terminus of SREBP-1 through the 2 × T7 tag or the C terminus through a 3xhemagglutinin (HA) Tag, which was substituted for the E-tag. For site 1, HA immunoprecipitation followed by trypsin digestion, which cleaves peptides after Arg or Lys, results in a peptide starting at Thr 319 (TILATVPLVVDAEK) (Fig. S5*A*), establishing cleavage by Nsp3 after Gly 318. For site 2, T7 immunoprecipitation followed by Asp-N digestion, which cleaves before Asp, resulted in a peptide terminating at Gly 341 (DAEKLPINRLAAG) (Fig. S5*B*). These findings provide strong independent confirmation of our mutagenesis data by mass spectrometry.

To determine whether the noncanonical sites we identified are responsible for the loss of the high-molecular weight form of SREBP-1 observed by CHX chase in Figure 2, we mutated site 1 and site 2 together and assessed SREBP-1 stability. Mutation of these two sites stabilizes SREBP-1 (Fig. 4*E* compare lanes 1 and 2 to lanes 5 and 6). Thus, sites 1 and site 2 are responsible for the significant Nsp3-dependent loss of SREBP-1 observed by CHX chase.

### Nsp3 stabilizes SREBP-2 by cleaving a C-terminal degron

**Figure 5:**
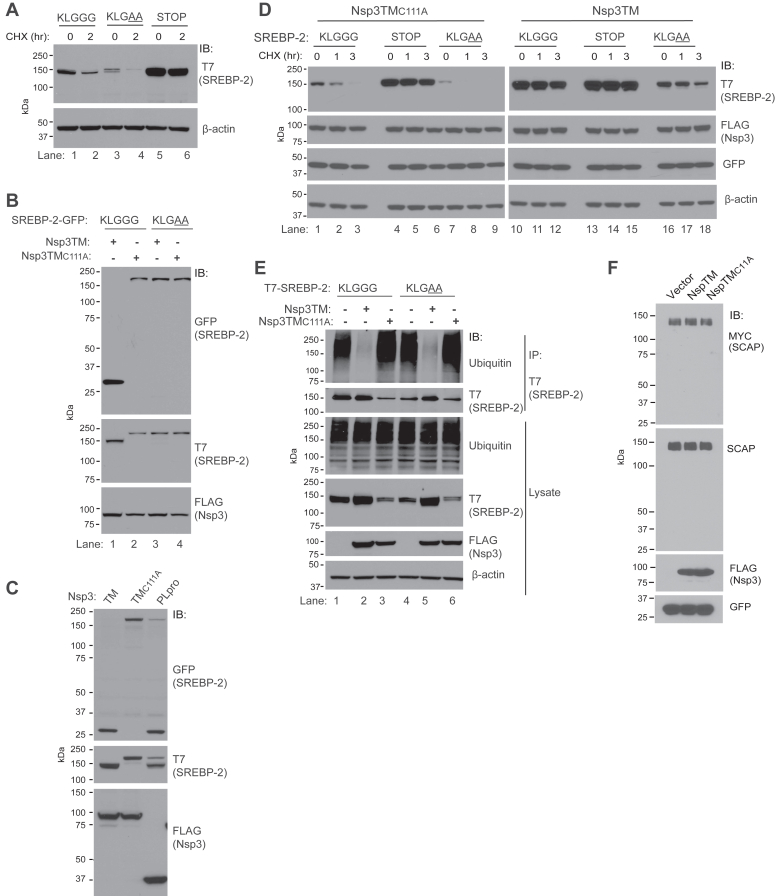
**Nsp3 increases SREBP-2 stability.***A*, HEK293T cells transfected with either WT SREBP-2 (KLGGG), a double mutation of Gly 1134 and 1135 to Ala (KLGAA), or a truncation after Gly 1135 (STOP) were assessed by CHX chase. *B*, C-terminal GFP fusions of WT SREBP-2 or the double Ala mutant were cotransfected with the indicated forms of Nsp3 in HEK293T cells and immunoblotted as indicated. *C*, GFP fusion of WT SREBP-2 was cotransfected with the indicated forms of Nsp3 and assessed as in (*B*). *D*, SREBP-2 plasmids described in (*A*) were cotransfected with WT or mutant Nsp3TM in HEK293T cells and levels assessed over a 3 h CHX chase. Samples on the *left* and *right* are identical exposures of material run on two gels that were transferred and evaluated simultaneously (see Fig. S6*D* for KLGAA samples run side-by side). Cells were split after transfection, β-actin serves as an equal loading control. *E*, HEK293T were transfected with the indicated forms of SREBP-2 and of Nsp3TM together with HA-ubiquitin. T7 immunoprecipitates were assessed for ubiquitin and SREBP-2 after treatment with MG132 for 3 h to allow for accumulation of ubiquitinated protein. Equal amounts of cell lysate were evaluated to ensure that changes in ubiquitination in immunoprecipitates did not reflect altered total cellular ubiquitination. *F*, cells transfected with C-terminal MYC epitope-tagged SCAP and the indicated forms of Nsp3 or vector control were treated with 20 μM MG132 for 4 h prior to lysis. Immunoblotting was with anti-MYC or with a SCAP antibody directed against a region N terminal to the LGGG sequence. Cotransfected GFP serves as transfection efficiency control. CHX, cycloheximide; Nsp, nonstructural protein; SREBP, sterol regulatory element–binding protein; SCAP, SREBP cleavage–activating protein; TM, transmembrane.

A reasonable hypothesis is that the SREBP-2 stabilization seen on overexpression in HEK293T cells in Figure 2, *E* and *F* and also observed in HeLa cells (Fig. S6*A*) is a consequence of Nsp3 DUB activity. However, it was recently reported that SREBP-2 has a discontinuous C-terminal degron, which is unmasked by dissociation from SCAP, allowing it to be targeted for proteasomal degradation by an unidentified ubiquitin ligase (48). This degron, consisting of aa 1129-1131 (IVK) and 1138-1141 (IAAS) at the C terminus of the protein (^1129^IVKLGGGTAIAAS∗), would target the normally stable SREBP-2 (49) for degradation, when present in excess relative to SCAP, and play a critical homeostatic role in eliminating the SREBP-2 C-terminal domain (CTD) after cleavage of the active transcription factor in the Golgi (48). Notably, beginning with 1131, in the middle of the degron, there is a sequence (KLGGG) that resembles the canonical site 3 sequence of SREBP-1 (RLGGG). We therefore assessed, by insertion of nonsense codons after KLGGG, whether simulating cleavage at this position stabilizes SREBP-2 (Fig. 5*A*, STOP). This truncation results in a marked stabilization of SREBP-2 (Fig. 5*A*). Mutation to KLGAA, which should prevent Nsp3-dependent cleavage of SREBP-2, did not result in SREBP-2 stabilization. These results confirm the importance of the C-terminal degron and suggest how Nsp3 could stabilize SREBP-2 by cleaving site 3 to interrupt the degron.

To establish whether Nsp3 interrupts the degron, we generated C-terminal GFP fusions of SREBP-2. For WT SREBP-2, coexpression of Nsp3TM, but not Nsp3TM_C111A_, results in a ∼30 kDa GFP-immunoreactive band with loss of the full-length fusion protein. This is not observed with the KLGAA mutation (Fig. 5*B*, compare lanes 1 and 3). Notably, cytosolic Nsp3PLpro can also cleave the C terminus of SREBP-2 (Fig. 5*C*). The finding of SREBP-2 cleavage by soluble PLpro correlates with the SREBP-2 stabilization observed in Figure 2*F*. Consistent with this cleavage, SREBP-2 coimmunoprecipitates with PLpro (Fig. S6*B*), although some coimmunoprecipitation of SREBP-1 is also observed with PLpro (Fig. S6*C*), despite the lack of SREBP-1 cleavage by cytosolic PLpro.

To further evaluate the effect of Nsp3 on SREBP-2, we assessed WT SREBP-2 (KLGGG), the noncleavable form (KLGAA), and the form terminating after KLGGG (STOP), by CHX chase. Nsp3TM results in a dramatic increase in the level and stability of the WT protein, similar to what is observed with the STOP mutation (Fig. 5*D*). However, even with the KLGAA mutation, where the C-terminal degron can no longer be interrupted, Nsp3TM still has a stabilizing effect (Fig. 5*D*, compare lanes 7–9 to 16–18, see also Fig. S6*D*). Importantly, this requires the catalytic activity of Nsp3TM. This result suggests that Nsp3 may not only be interrupting a degron by cleavage at a canonical site but also has the potential to decrease degradation of SREBP-2 by deubiquitination when the degron is mutated or inaccessible. To assess this, we examined SREBP-2 ubiquitination. When the degron is intact and the protein is normally targeted for ubiquitin-dependent degradation, significant accumulated ubiquitination is observed when proteasomal degradation is inhibited. Nsp3TM results in a loss of ubiquitination as would be expected with inactivation of the degron by PLpro cleavage (Fig. 5*E* upper panel, lanes 1–3). However, even with the KLGAA mutation, where the degron is no longer cleavable, a marked decrease in ubiquitination is observed with Nsp3TM (Fig. 5*E* lanes 4–6), which correlates with stabilization of this mutation (Figs. 5*D* and S6*D*). Thus, Nsp3 can prevent SREBP-2 degradation through two mechanisms: it functions as an endoprotease to remove the degron and can potentially function as a DUB to facilitate SREBP-2 deubiquitination.

### Human SCAP contains a canonical Nsp3 cleavage site but shows no evidence of cleavage

Our findings raise the possibility that cleavage at LXGG by the PLpro of Nsp3 might be widespread in SARS-CoV-2–infected cells, particularly in the secretory pathway. Human SCAP, but not the extensively studied hamster SCAP (50), contains an LGGG sequence (aa 741–745) in its cytoplasmic domain. After confirming previous observations that SCAP shows ER localization when overexpressed (51) (Fig. S6*E*), we evaluated it for cleavage when coexpressed with Nsp3TM. We observe no loss of the 145 kDa full-length species or appearance of additional fragments corresponding to N- or C-terminal halves of the protein when coexpressed with Nsp3TM (Fig. 5*F*). While we cannot exclude that SCAP may be an Nsp3 substrate in the context of SARS-CoV-2 infection, we see no evidence of cleavage under conditions where efficient cleavage of analogous sequences in SREBP-1 and SREBP-2 is observed.

## Discussion

In this study, we demonstrate that SARS-CoV-2 PLpro, when expressed as a transmembrane protein, can function as a deubiquitinating enzyme for some potential ERAD substrates. Moreover, our findings establish the capacity of transmembrane forms of SARS-CoV-2 PLpro to cleave cellular ER proteins at both canonical and noncanonical sites. Most of what we observe requires PLpro to be tethered to the ER membrane. Strikingly, for canonical sites that are similarly positioned within the related SREBP-1 and SREBP-2, a requirement for membrane anchoring of PLpro was found for the former but not the latter. Our observations emphasize the importance of evaluating SARS-CoV-2 PLpro in its transmembrane ER form. They also demonstrate the need for empirical observation to identify cleavage of novel substrates, including at unpredicted sites.

### Canonical and noncanonical Nsp3 cleavage and context-dependent activity

Cleavage of SREBP-1 at site 1 and site 2, although noncanonical and exhibiting incomplete cleavage relative to site 3, is sufficient to diminish levels of exogenously expressed SREBP-1 having an intact NTD transcription factor precursor. Val, at the P_4_ position of site 1 (LVSGG), has not been reported as being a substitute for Leu in this position. However, Ala substitutions within the canonical PLpro cleavage sites in the SARS-CoV-1 polyprotein revealed that there is a capacity for a residue other than Leu to allow for efficient cutting uniquely at the Nsp2-Nsp3 cleavage site (8). In our case, substitution of Ala for Val largely eliminates cutting, although a small amount is observed on long exposures (Fig. 4*D*). The finding that, in certain contexts, residues other than Leu in P_4_ still permits cutting by PLpro is consistent with structural analysis of SARS-CoV-2 PLpro bound to enzyme inhibitors, which suggests flexibility in the region that recognizes this residue (19). A more striking finding comes from SREBP-1 site 2, which has an Ala rather than a Gly in the P_2_ position (RLAAG), and where substitution with either Leu or Glu inactivates this site. Yet, substitution of Ala for Gly in the P_2_ position of either site 1 (LVSGG) or site 3 (RLGGG) results in the loss of detectable cutting.

There have been few studies examining cellular proteins that can be cleaved by PLpro. These studies have utilized the isolated PLpro and have not identified noncanonical sites or assessed site-specific requirements for cleavage. A recent study selectively assessed a region of CD177, containing a canonical PLpro consensus sequence, and found it could be cleaved by SARS-CoV-2 PLpro *in vitro* when expressed by phage display (52). A study using cytosolic SARS-CoV-2 PLpro and an MS approach uncovered several “neo-N termini” from proteins having canonical sites (53). In another study, Moustaqil *et al*. (5) evaluated 71 human innate immune pathway proteins using recombinant proteins and cell lysates incubated with the SARS-CoV-2 PLpro. Of these, interferon regulatory factor 3, which includes an LGGG, was cleaved by PLpro while three other proteins having canonical sequences were not. This specificity is consistent with our observation that SCAP, despite having a cytoplasmic LGGG, is not cleaved by the protease. Selectivity in substrate cleavage may be due in part to protein folding. However, the finding of noncanonical cleavage in some sites but not others (*e.g.*, P_2_ Ala in site 2), and differential activity of soluble PLpro toward similar sites on SREBP-1 and SREBP-2, underscore the importance of context in PLpro activity toward cellular substrates.

### Complexities and potential implications of INSIG and SREBP regulation

Our findings offer a glimpse into alterations in ER protein fate and function that can occur with SARS-CoV-2 infection. These results, employing individual ER proteins coexpressed with different forms of PLpro, allow for insights into the potential for PLpro to either deubiquitinate ERAD substrates or cleave proteins that enter the secretory pathway. However, for any individual protein, identified in this study or elsewhere, its fate will be influenced by the cellular milieu of SARS-CoV-2 infection. In considering just the secretory pathway, viral infection results in changes that include dissolution of the Golgi, condensation of the ER, and partitioning of ER membrane into the replication/transcription complex (2, 3, 4). These undoubtedly affect membrane proteins and their interactions including the accessibility of SREBP-1 and SREBP-2 to activating Golgi proteases, as well as potentially affecting interactions with SCAP, thus impacting pathways and transcriptional feedback loops that characterize lipid biogenesis. Similarly, other intricately regulated processes such as ERAD and ER stress responses will be affected in ways that are not readily predictable.

There are, though, intriguing possibilities related to SREBPs and the relationship between lipid biogenesis and the innate immune response to infection that potentially arise from our findings should they also occur in infected cells. In macrophages, diminished SREBP-2 expression is associated with decreased ER membrane cholesterol, which facilitates the type 1 IFN response (54, 55). Stabilization of SREBP-2 by Nsp3 could potentially contribute to the attenuation of the immune response seen with SARS-CoV infection. The stabilization of SREBP-2, and particularly the residual CTD, which occurs with disruption of the degron, has been shown by Kober *et al*. to decrease SCAP available to transport SREBPs to the Golgi (48). Interestingly, SCAP has been reported to bind STING and interferon regulatory factor and facilitate the IFN response by translocating to perinuclear microsomes in response to microbial DNAs (51). Thus, should Nsp3 increase the stability of SREBP-2 or of its CTD during SARS-CoV infection, it might also diminish the pool of SCAP available for this step in innate immune response activation. Finally, stabilization of SREBP-2 or its CTD would provide a molecular basis for the provocative finding that increased serum levels of the SREBP-2 CTD correlate with the clinical severity of COVID-19 by contributing to cytokine storm and pulmonary damage (56).

## Experimental procedures

### Cells

HEK293T and HeLa cells, obtained from ATCC, were maintained in Dulbecco's modified Eagle's medium supplemented with 10% (v/v) fetal bovine serum, 100 units/ml penicillin, 100 μg/ml streptomycin, and 2 mM glutamine in a humidified incubator at 37 °C and 5% CO_2_. Cells used in experiments were routinely tested for *Mycoplasma* using LookOut *Mycoplasma* PCR Detection Kit (Sigma-Aldrich) and MycoStrip *Mycoplasma* Detection Kit (Invivogen) and found to be negative.

### Plasmid constructs

Complementary DNA encoding the full-length Nsp3 protein from the SARS-CoV-2 isolate Wuhan-Hu-1 (https://www.addgene.org/141257/), obtained from Addgene, was a gift from Dr Fritz Roth. The full-length protein with an N-terminal 3× FLAG epitope in CMV5.1p was generated by recombination using Gateway system (Invitrogen). Plasmids encoding Nsp3 beginning at aa743, preceding the beginning of the PLpro (aa 746) and terminating either following an amphipathic helix that is immediately C terminal to the second predicted transmembrane domain at aa 1579 (Nsp3TM) or following the PLpro domain at aa 1065 (Nsp3PLpro), were generated using In Fusion (Takara) and cloned into the single N-terminal FLAG epitope–containing pCMV-Tag2B vector from BamHI to EcoRI. Mutation of the catalytic Cys to Ala (aa 856 of Nsp3; aa 111 defined by the beginning of the PLpro) was carried out by site-specific mutagenesis (QuickChange II, Agilent).

A cDNA encoding SREBP-1 isoform 1 with a 2 × T7 tag at its N terminus and an E-tag at its C terminus and silent mutations and mutations in regions critical for S1P and S2P cleavage was synthesized by GenScript and cloned into pCDNA3.1(+) from NheI to NotI (GenScript project ID: U124AFG200-5). pCDNA3.1(+) T7x2-SREBP-1-E with WT sequence, used in this study, was then generated by substituting a cDNA fragment synthesized by Integrated DNA Technologies using designed internal HindIII and XbaI sites. T7x2-SREBP-1-HA was generated by synthesis of a cDNA fragment encoding His_8_ and HAx3 in pUC57 by GenScript and cloning into T7x2-SREBP-1-E from EcoRI to NotI, which resulted in substitution of this sequence for the E-tag. To generate T7x2-SREBP-1-HA-GFP, eGFP was amplified from pEGFP-C1 (Clontech), first cloned into the pUC57 construct from KpnI to NotI, substituting GFP for His_8_, and then into T7x2-SREBP-1-E from EcoRI to NotI. A construct encoding aa 1 to 520 of SREBP-1, approximating the N-terminal active transcription factor, was generated by amplifying a region corresponding to aa 410 to 520 of T7x2-SREBP-1-E and cloning this into U124AFG200-5 from HindIII to XbaI. SREBP-2 with a 3 × T7 tag at its N terminus (T7x3-SREBP-2) was synthesized by GenScript and cloned into pcDNA3.1(+)-N-HA from ClaI to NotI. GFP was inserted by subcloning a fragment beginning with an internal EcoRI site in SREBP-2 to a NotI site in the C-terminal polylinker. The eGFP was generated by PCR and first subcloned into a shuttle vector from EcoRI to XbaI. All mutations, internal deletions, and truncations in SREBP-1 and SREBP-2, except as mentioned above, were generated by site-specific mutagenesis and confirmed by sequencing.

Plasmid encoding INSIG-1-MYC having a 6xMYC tag, HA-NHK, and concatemerized HA-Ub were gifts from Joseph Goldstein, Ron Kopito, and Dirk Bohmann, respectively (43, 50, 57). HA-tagged human Ub (single copy) was synthesized by Integrated DNA Technologies and subcloned into pcDNA3.1(+) between BamHI and EcoRI with a linker of ASASRP. Plasmid encoding human SCAP in pCMV6-entry with C-terminal MYC and FLAG tags was obtained from OriGene (CAT#: RC223734) and sequenced in full. A single polymorphism relative to the reference sequence (UniProtKB/Swiss-Prot: Q12770.4) was noted at position 798 (Val to Ala) of the plasmid from Origene, within the predicted cytoplasmic domain of the protein. Two stop codons were inserted after the MYC tag to remove the FLAG tag, which was also sequenced.

### Antibodies

FLAG immunoprecipitations were with either anti-FLAG M2 Affinity Gel Beads (Sigma A222) or anti-FLAG M2 Magnetic Beads (Millipore Sigma M8823). In some experiments, normal mouse IgG (Santa Cruz Biotechnology #sc-2025) was used as a control bound to protein G MagBeads (GenScript L00274). MYC epitope immunoprecipitation was with either EZview Red anti-c-Myc Affinity Gel (Millipore Sigma E6654) or anti-FLAG M2 Magnetic Beads (Millipore Sigma M8823). FLAG immunoblots were with either monoclonal ANTI-FLAG M2-Peroxidase (HRP) produced in mouse (Sigma A8592) or goat anti-FLAG HRP (Bethyl Laboratories A190-101P). T7 immunoblots were with goat anti-T7 horseradish peroxidase (HRP) (Bethyl Laboratories A190-116P). E-tag immunoblots were with goat anti-E-tag HRP (Bethyl Laboratories A190-132P). MYC immunoblots were with 9E10 (36) or MYC-tag 71D10 (Cell Signaling Technology 2278). HA immunoblots were with HRP-conjugated rat anti-HA (Clone 3F10, Roche). GFP antibodies used were GFP antibody B-2 (Santa Cruz Biotechnology sc-9996) and rat anti-GFP 3H9 (Chromotek 3h9-100). Immunoblotting for β-actin was with Millipore Sigma A5441. Ubiquitin antibody has been described (58). Rabbit antibody directed against human SCAP was from Cell Signaling Technology (13102). SREBP-1 mAb 2A4 (47) was from Novus (NB600-582). Species-specific secondary antibodies for immunoblotting were from GE Healthcare (NA931, NA934) or Jackson Laboratories (115-035-174, 211-032-171). Primary and secondary antibodies used for immunofluorescence for Calnexin colocalization with Nsp3 and SCAP include the following: rabbit polyclonal Calnexin (Proteintech 10427-2-AP), goat anti-rabbit-488 (Invitrogen A10521), FLAG M2 (Millipore Sigma F1804), and goat anti-Mouse IgG (H&L)—Alexa Fluor 568 (Abcam ab1754). For colocalization of SREBPs with Nsp3TM, reagents include the following: mouse anti-T7 (Novagen 69522), goat anti-mouse Cy3 (Invitrogen A10521), rabbit anti-FLAG (Cell Signaling Technology D6W5B), and goat anti-Rb-488 (Invitrogen A10521).

### Cell experiments

Cells were transfected with the indicated plasmids for 30 to 40 h using either PolyFect (Qiagen) or JetPrime (Polyplus 1010000046). For assessment of protein levels and protein loss, cells were lysed either immediately or at the indicated times in lysis buffer 1: PBS pH 7.4, 1% Triton X-100, 0.5% sodium deoxycholate (INSIG-1 and NHK) or in lysis buffer 2: 10 mM Tris–HCL pH 8, 140 mM NaCl, 1% Triton X-100, 0.1% SDS (SREBP-1, SREBP-2, and SCAP), supplemented with 1 mM EDTA and phosphatase inhibitor (APE × BIO, K1014). Lysis buffers were supplemented with 20 to 40 μM MG132 (APE × BIO A2528), 10 mM iodoacetamide, and cOmplete Protease Inhibitor Cocktail EDTA-free (Roche 11836153001). Insoluble material was removed by centrifugation at >15,000×*g* for 5 to 10 min. Equal loading of protein was determined by Bradford assay. For CHX chase experiments, CHX was added to a final concentration of 50 μg/ml. In some experiments, samples were split into individual wells following transfection and β-actin served as loading control; otherwise, equal transfection efficiency was assessed using cotransfected eGFP. To assess requirements for SREBP-1 and SREBP-2 cleavage (experiments not involving CHX), cells were treated with MG132 (40 μM) for either 2 h (HEK293T) or 4 h (HeLA) prior to harvesting. For assessment of SCAP in HEK293T cells, treatment was also for 4 h. For immunoprecipitation experiments, cells were pretreated with MG132 either overnight (10 μM) or for 3 h (25 μM) prior to lysis in lysis buffer 2. After removing insoluble material by centrifugation, the clarified lysates were used for immunoprecipitation at 4 °C for 2 to 4 h, and the beads were washed three times with 10× bed volumes of lysis buffer 2. For phosphatase treatment, cells were lysed in lysis buffer 2 without EDTA or phosphatase inhibitors and incubated with 400 units of Lamba Protein Phosphatase (New England Biolabs P0753S) in a final volume of 50 μl of 1× PMP buffer and 1 mM MnCl_2_ (New England BioLabs kit P0753S) at 30 °C for 45 min. For assessment of INSIG-1 ubiquitination, cells were transfected with INSIG-1-MYC and HA-tagged ubiquitin. Cells were lysed in 1% SDS, heated for 1 min, and diluted with lysis buffer 1. The clarified lysate was used for immunoprecipitation, and ubiquitination was detected using anti-HA. For assessment of SREBP-2 ubiquitination, cells were transfected with T7-tagged SREBP-2 and HA-tagged ubiquitin. Cells were lysed in lysis buffer 2 for immunoprecipitation and ubiquitin detected with ubiquitin antibody.

Immunoprecipitates and clarified cell lysates were denatured in reducing lithium dodecyl sulfate sample buffer (Thermo Fisher Scientific NP008), followed by resolution by SDS-PAGE and transfer to nitrocellulose membranes (Thermo Fisher Scientific 88018). Immunoblots were developed either using primary antibodies followed by HRP-conjugated secondary antibodies or primary antibodies directly conjugated to HRP followed by enhanced chemiluminescence using SuperSignal West Pico Plus (Thermo Fisher Scientific 1863096) or Clarity Western ECL (Bio-Rad 170-5061). Images were obtained either on films or by using an Azure Biosystems c280 imager.

### Mass spectrometry

HEK293T cells, grown in 10 cm dishes, were cotransfected with plasmid encoding Nsp3TM and T7x2-SREBP-1-HA having a mutated site 3 (KLGGA) and either a mutated site 1 (LVSGA) or site 2 (RLAAA). After 24 h, samples were treated with 40 μM MG132 for 2 h, lysed in lysis buffer 2, and soluble material immunoprecipitated sequentially with T7 agarose beads (EMD Millipore), followed by Pierce Anti-HA Magnetic Beads (Thermo Fisher Scientific).

After extensive washing, beads were resuspended in 25 mM Hepes, pH 8 and heated at 95 °C for 5 min to denature proteins. The samples were digested overnight with either 2 μg of trypsin (Promega) or 2 μg of Asp-N (Promega) at 37 °C. The supernatants containing protease-digested peptides were collected after centrifugation of beads and the peptides cleaned with the EasyPep micro sample preparation kit (Thermo Fisher Scientific, Cat# A57864) and lyophilized.

The peptides were resuspended in 0.1% TFA and subjected to nanoflow liquid chromatography (Thermo Easy nLC 1200, Thermo Fisher Scientific) coupled to high-resolution tandem MS (Orbitrap Lumos, Thermo Fisher Scientific). MS scans were performed in the Orbitrap analyzer at a resolution of 120,000 with an ion accumulation target set at 4e^6^ over a mass range of 400 to 1600 *m/z*, followed by tandem mass spectrometry analysis at a resolution of 15,000 with an ion accumulation target set at 5e^5^. Fragment precursor isolation width was setup at 1.6 *m/z*, normalized collision energy was 30, and charge state 1 and unassigned charge states were excluded. Acquired MS/MS spectra were searched against human UniProt protein database using a SEQUEST and percolator validator algorithms in the Proteome Discoverer 2.4 software (Thermo Fisher Scientific) (https://www.thermofisher.com/us/en/home/industrial/mass-spectrometry/liquid-chromatography-mass-spectrometry-lc-ms/lc-ms-software/multi-omics-data-analysis/proteome-discoverer-software.html). Enzyme was specified as Trypsin (set as Semi tryptic) or Asp-N. The precursor ion tolerance was set at 10 ppm, and the fragment ions tolerance was set at 0.02 Da along with methionine oxidation as dynamic modification. A false discovery of 0.01 and a minimum peptide length of six amino acids were used for peptide identification.

### Fluorescence microscopy

Cells were plated at a confluence of ∼60% into 12-well plates containing 18 mm diameter cover slips (Ted Pella 26022) that had been pretreated with 50 mM poly-D-lysine (Sigma P1024). After 5 h, cells were transfected with indicated plasmids using JetPrime. After 24 h, cells were fixed for 10 min in 4% paraformaldehyde at room temperature and permeabilized for 5 min in 100% methanol precooled to −30 °C. The coverslips were then washed with PBS pH 7.4 containing 0.05% Tween 20 (PBST) and treated with buffer F (PBST supplemented with 5% goat serum) for 1 h to reduce nonspecific absorption. After washing three times with PBST, coverslips were incubated with the indicated primary antibodies in buffer F for 1 h at room temperature, washed three times with PBST, and then incubated with secondary antibodies for 1 h at room temperature. After washing three times with PBST, coverslips were mounted onto a glass slide in mounting medium (90% glycerol in 1M Tris–HCl pH 8.5, 1 mg/ml p-Phenylenediamine) for imaging.

Widefield images were acquired using inverted Eclipse Ti2 inverted microscope (Nikon Inc), equipped with or an ORCA-Flash4.0 V3 (Hamamatsu), Lumencor SPECTRA light engine (Lumencor Inc), 100× NA 1.42 and 1.45 Plan Apo objective, and 1.5× magnifying tube lens. 200-nm thick Z sections were acquired.

Structured illumination microscopy (SIM) was performed on N-SIM (Nikon Inc), equipped with 405, 488, 561, and 640 nm excitation lasers, Apo TIRF 100× NA 1.49 Plan Apo oil objective, and back-illuminated 16 μm pixel EMCCD camera (Andor, DU897). 100-nm thick Z Sections were acquired in 3D SIM mode and reconstructed. Fiji (National Institutes of Health) (https://fiji.sc/) and Photoshop (Adobe) were used to assemble image panels. AutoQuant X3 software (MediaCybernetics) (https://www.meyerinst.com/mediacybernetics/autoquant/) was used for deconvolution. Image panels show one representative Z plane.

To illustrate the typical pattern of signal overlap from microscopy images, line plots were drawn across multicolor images as indicated in the figure panels. The plot profile function in Fiji (ImageJ2) was used to determine the intensities of the fluorescent signals along the drawn line. The data was exported to Prism (GraphPad) (https://www.graphpad.com/) to generate plot profiles.

## Data availability

MS data is available on the MassIVE server reference number: MSV000092963. All other data are contained within the manuscript.

## Supporting information

This article contains supporting information.

## Conflict of interest

The authors declare that they have no conflicts of interest with the contents of this article.
