# Supplementary material for: SARS-CoV-2 papain-like protease plays multiple roles in regulating cellular proteins in the endoplasmic reticulum
Source: J Biol Chem. 2023 Oct 12;299(12):105346. doi: 10.1016/j.jbc.2023.105346 (PMC10692909; doi:10.1016/j.jbc.2023.105346)
Supplement: Supporting information [file mmc1.pdf]

Supporting Information for:

SARS-CoV-2 papain-like protease (PLpro) plays multiple roles in regulating cellular proteins in the endoplasmic reticulum

Mei Yang[1], Jennifer Mariano[1], Rebecca Su[1], Christopher E. Smith[1#], Sudipto Das[2], Catherine Gill[1], Thorkell Andresson[2], Jadranka Loncarek[1], Yien Che Tsai[1], Allan M. Weissman[1*]

1. Cancer Innovation Laboratory, Center for Cancer Research, National Institutes of Health, Frederick Maryland, 21702

2. Protein Characterization Laboratory, Cancer Research Technology Program, Frederick National Laboratory for Cancer Research, Frederick, Maryland, 21701

# Current address: One Amgen Center Dr., Thousand Oaks, CA 91320

*Corresponding author
Email: weissmaa@mail.nih.gov

Contents: Supporting Figures 1-6

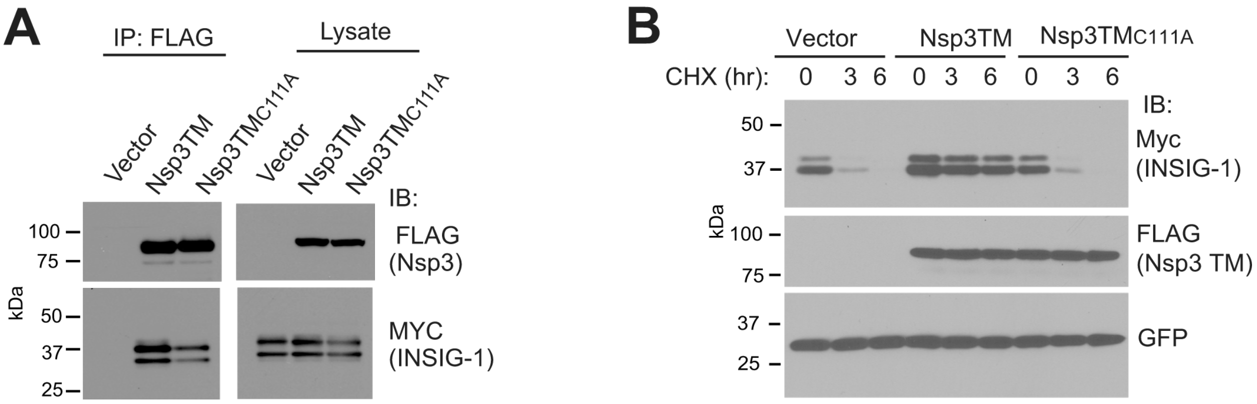

Figure S1. *A,* HEK293T cells were transfected with the indicated forms of Nsp3 or vector control, along with INSIG-1. Equal amounts of lysates from each of the MG132-treated samples were immunoprecipitated with FLAG (Nsp3) and immunoblotted to assess co-immunoprecipitation, equal amounts of whole cell lysate are shown to demonstrate relative protein levels. *B*, HeLa cells were transfected with C-terminally MYC-tagged INSIG-1 and either WT or mutant Nsp3TM or vector control and assessed by cycloheximide (CHX) chase.

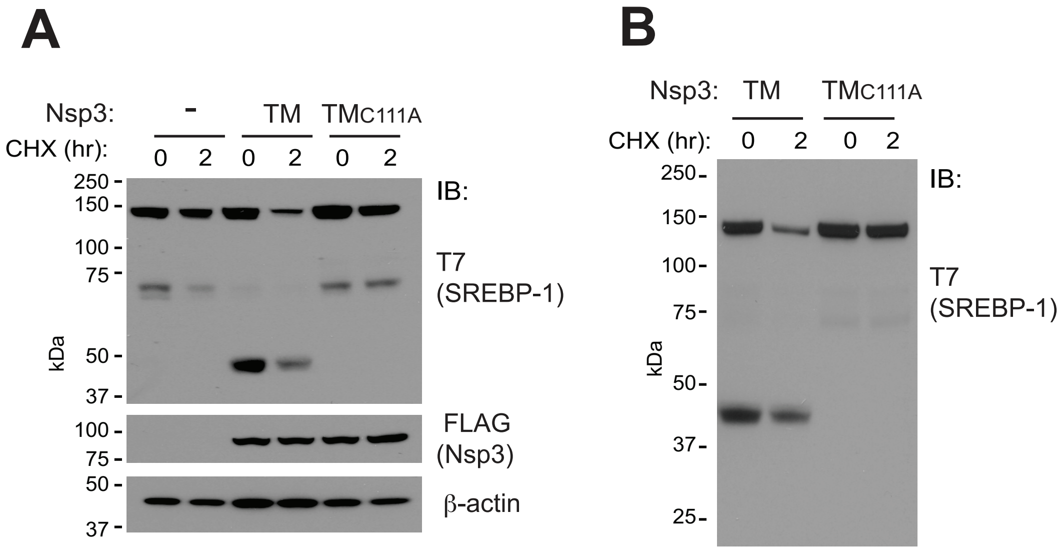

Figure S2. *A,* HEK293T Cells were transfected with T7-tagged SREBP-1 lacking the C-terminal E-tag along with the indicated forms of Nsp3 or vector control (-) and assessed by immunoblotting for levels and degradation after two hours of CHX. *B*, Positive control for Figure 2D demonstrating cleavage of SREBP-1 in the same experiment.

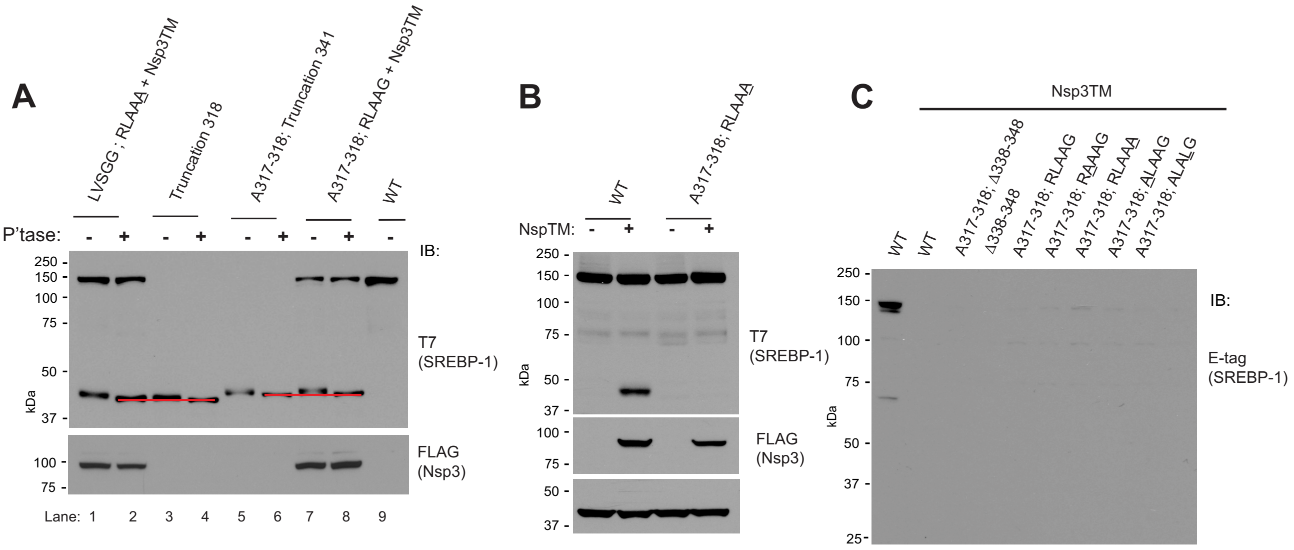

Figure S3. *A*, HEK293T cells were transfected with either full length SREBP-1 having only Site 1 (lanes 1 and 2) or Site 2 (lanes 7 and 8) intact, together with Nsp3TM, alternatively they were transfected with truncations at aa318 (lanes 3 and 4) or at aa341 (lanes 7 and 8) in a construct containing the identical site 1 mutation used in lanes 5 and 6. Samples were either treated or not with phosphatase (P'tase) before resolving by SDS-PAGE. WT SREBP-1 alone is transfected in lane 9. Red lines illustrate co-migration of phosphatase-treated SREBP-1, having either Site 1 or Site 2 intact, with the corresponding phosphatase-treated truncations. *B,* HeLa cells were transfected with either WT SREBP-1 or a double mutation in Sites 1 and 2 and co-transfected with Nsp3TM or vector control (-). *C,* Complete E-tag autoradiograph from Figure 3D.

A

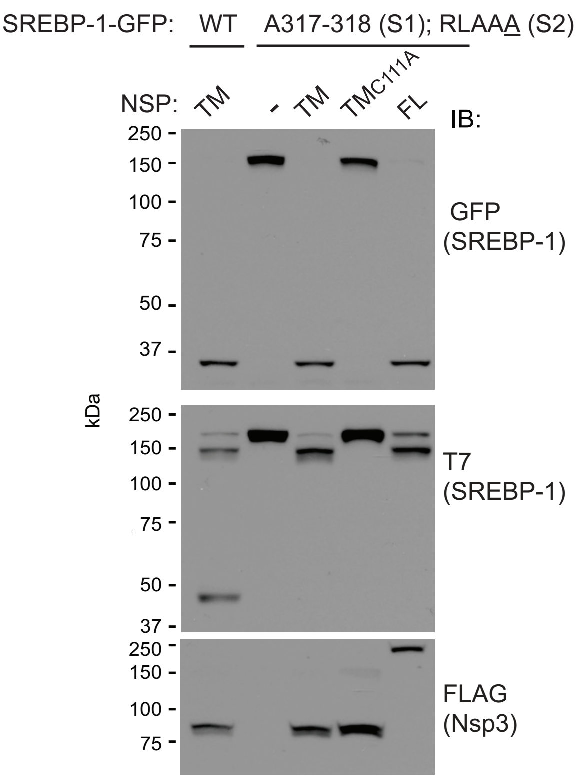

Figure S4. *A*, HEK293T cells were transfected with either WT SREBP1-GFP or a mutant in the first two cleavage sites and the indicated forms of Nsp3 or vector control (-) and immunoblotted as indicated.

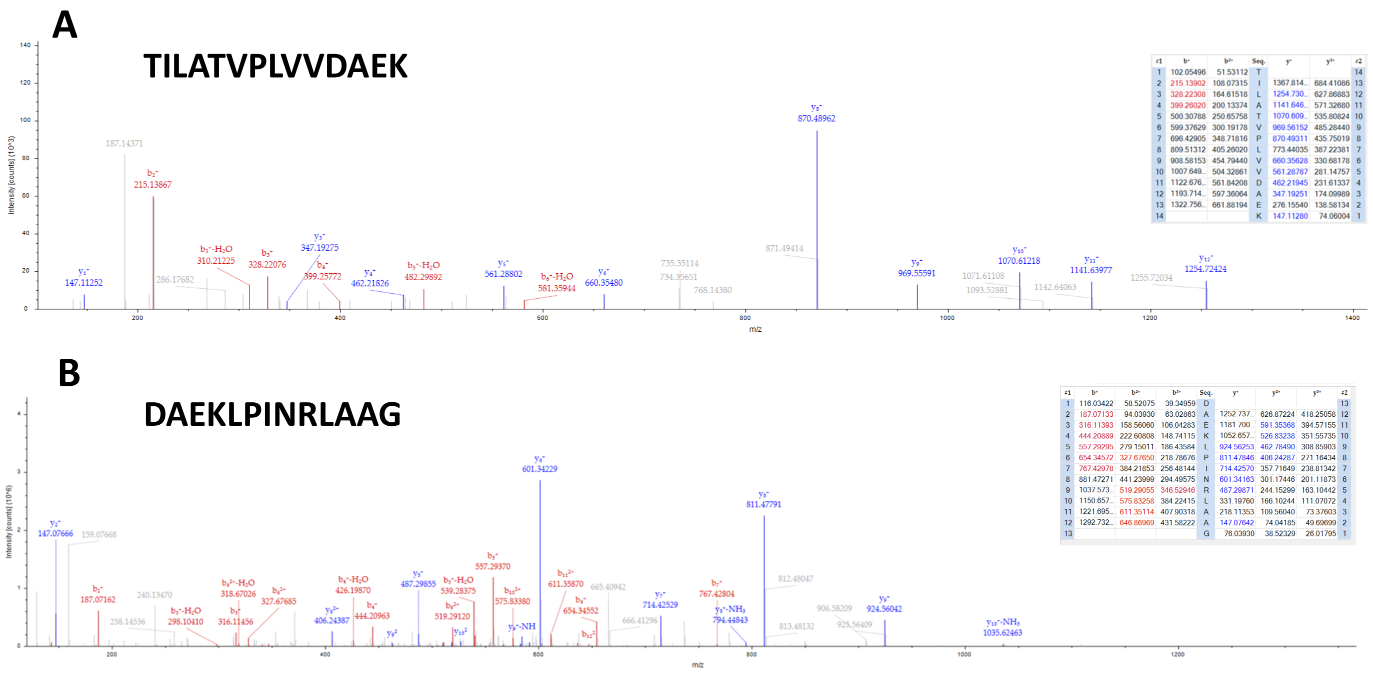

Figure S5. *A&B***,** Fragment spectra for two specific proteolytic peptides from the human SREBP-1 protein expressed in HEK293T cells demonstrating cleavage by Nsp3TM at two non-canonical sites within SREBP-1 in HEK293T cells. *A,* Mass spectrum for the **TILATVPLVVDAEK** peptide that is generated by trypsin only when the SREBP-1 has been cleaved by NSP3 C-terminal to Gly318 of Site 1 (LVSGG^TILATVPLVVDAEK). The table shows all b-ions (red) and y-ions (blue) that were detected during the fragment ion acquisition. *B,* Mass spectrum for the **DAEKLPINRLAAG** peptide that is generated by Asp-N only when the SREBP-1 has been cleaved by NSP3 at Gly341 of Site 2 (DAEKLPINRLAAG^). The table shows all b-ions (red) and y-ions (blue) that were detected during the fragment ion acquisition.

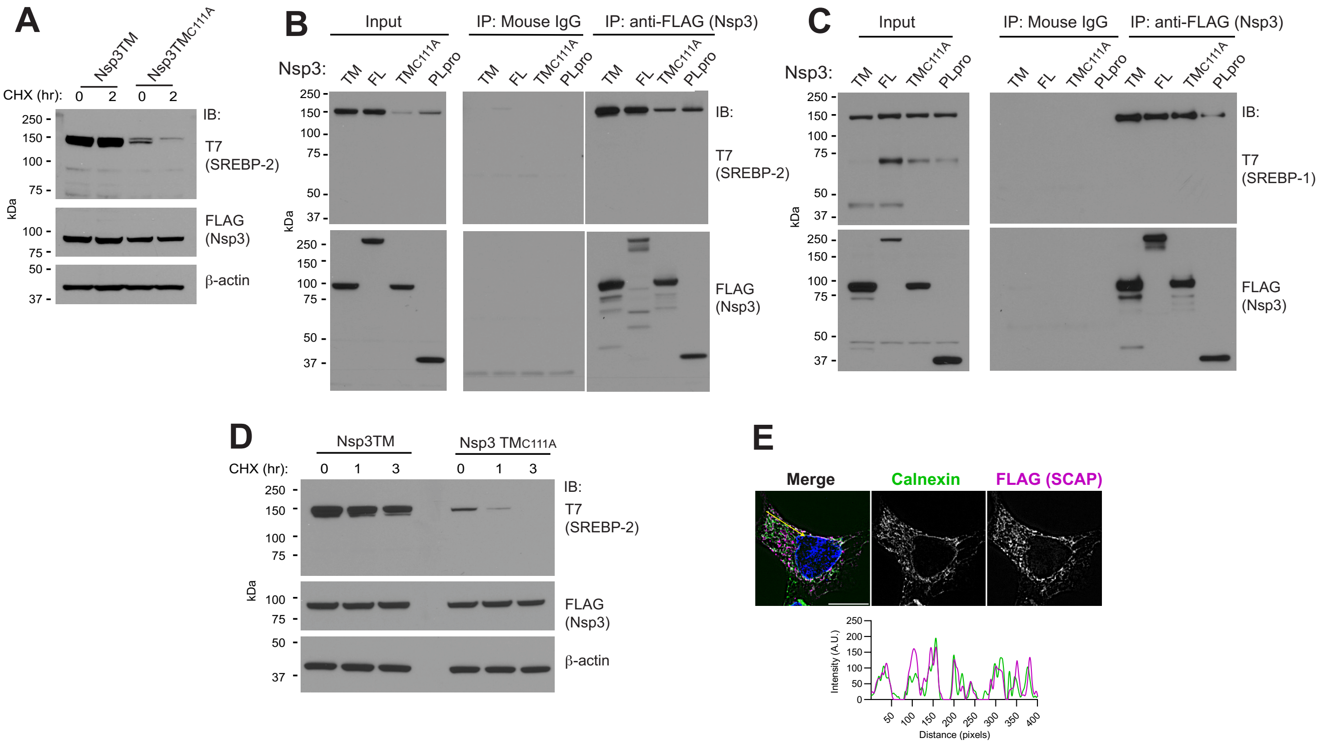

Figure S6. *A*, HeLa cells were transfected with SREBP-2 and either WT or mutant Nsp3TM and stability assessed by CHX chase. *B&C***,** HEK293T cells were transfected with SREBP-2 (*B*) or SREBP-1 (*C*) and the indicated forms of Nsp3 and treated with MG132 prior to harvesting. Equal amount of lysate from the same transfected sample was subject to immunoprecipitation with either FLAG antibody or mouse IgG, which serves as a negative internal control, and immunoblotted as indicated. Equal amounts of the four different lysates used for IPs are shown to the left. *D*, Material identical to that shown in Figure 5D for the SREBP-2 KLGAA mutation is shown run side-by-side. *E***,** HEK293T cells were transfected with SCAP C-terminally tagged with MYC and FLAG. Samples were assessed for co-localization with endogenous Calnexin. Scale bar corresponds to 10 μm. Assessment of co-localization is described in Figure 1B.
